# Scheduling management and optimization analysis of intermediate products transfer in a shipyard for cruise ships

**DOI:** 10.1371/journal.pone.0265047

**Published:** 2022-03-22

**Authors:** Jiajie Liu, Jingbo Yin, Rafi Ullah Khan

**Affiliations:** State Key Laboratory of Ocean Engineering, School of Naval Architecture, Ocean and Civil Engineering, Shanghai Jiao Tong University, Shanghai, China; Tongji University, CHINA

## Abstract

Shipbuilding is a complex and large-scale operation involving many intermediate products (blocks) and the frequent transfer of blocks among workshops and stockyards. The reasonable use of methods to complete the transfer scheduling of intermediate products is of great importance. In this paper, the blocks and the flat transporters are the research objects. Based on organizing the various logistical processes for blocks and the circulation process in the shipyard, we established a model that takes the task time window and other factors as constraints, and minimizes the sum of delay time and no-load time of flat transporters while satisfying the punctuality of scheduling tasks. Three conclusions are reached: (1)The flat transporter utilization rate is inversely related to the value of the objective function. The smaller the value of the objective function, the more the usage rate of a particular one (2) loading is the biggest obstacle to the overall working time of flat transporters, and a simple optimization model cannot solve this problem; and (3) based on the optimization model, the load efficiency of flat transporters can be improved, and the delivery time can be reduced.

## 1. Introduction

**Fig 1:**
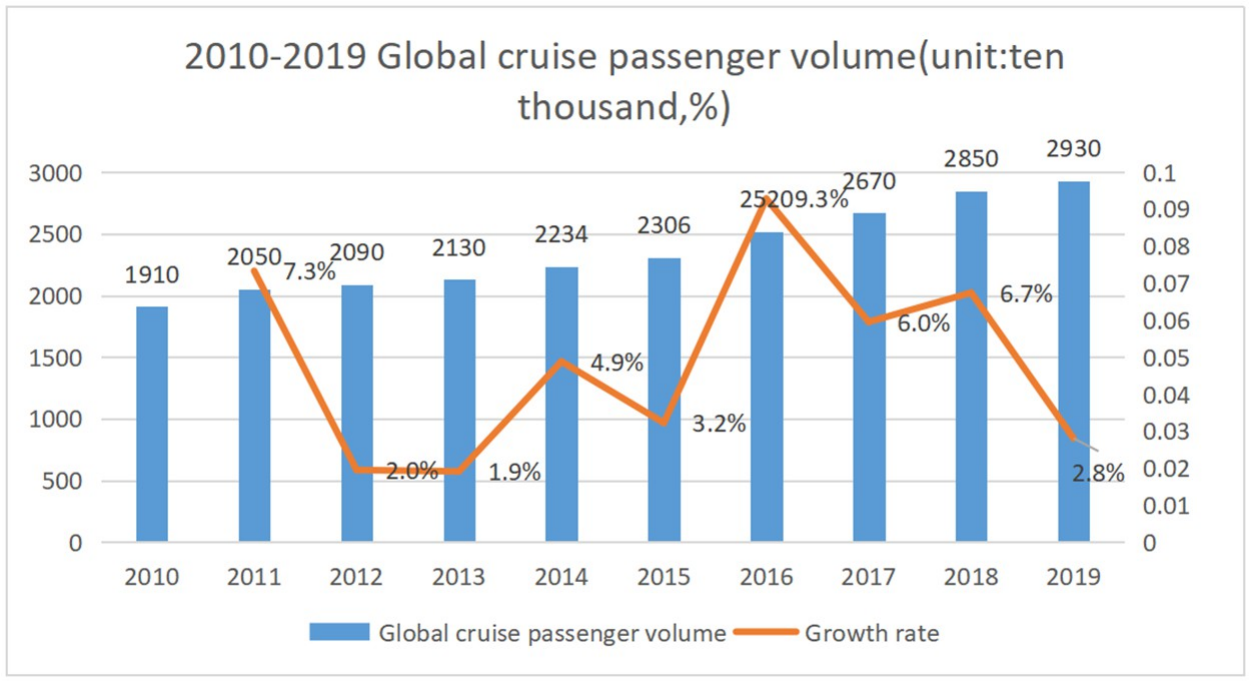
2010–2019 global cruise passenger volume.

Cruise ships are large luxury passenger ships that sail the oceans on dedicated routes over fixed periods of time. According to data released by the International Cruise Line Association, the number of global ocean cruise passengers continued to grow from 2010 to 2019 (as shown in Fig 1). The cruise market is getting larger, and more people are choosing cruise ships for their vacations. In 2019, the number of global cruise tourists reached a new high of about 29.3 million. Although the growth rate has slowed compared with the previous period, the overall development of the global cruise industry has bright prospects and investment potential.

The China Cruise Industry Development Report (2019) predicted that the scale of Chinas cruise market would reach 300 billion to 400 billion yuan in 2020. Although China is already the second-largest cruise market globally, the penetration rate of domestic cruise tourism is still less than 1%. Influenced by positive factors such as national policy support and consumption upgrading, this market has strong market potential.

Cruise ships are important cultural carriers on Chinas Maritime Silk Road and in other national strategies, and recent research has revealed that the Maritime Silk Road has influenced many areas and needs to be managed well [1]. The Made in China 2025 report issued by the State Council notes that the design and construction of high-tech ocean-going passenger ships is an important aspect of the development of Chinas shipbuilding industry. In July 2017, the Ministry of Industry and Information Technology issued the Guide to Scientific Research Projects of High-Tech Ships (2017 edition), which clearly indicated the need to make advancements in the engineering design and construction technology of high-tech ocean-going passenger ships and to raise standards and improve intellectual property logistics management systems for the construction of such vessels.

Due to the limited annual output of these ships globally, it is difficult for domestic enterprises to buy ready-made, second-hand luxury cruise ships that meet the needs of the Chinese market. Therefore, it is important that China masters the manufacturing technology required to build high-tech ocean-going passenger ships [2]. However, shipbuilding differs from other manufacturing industries. Products of the shipbuilding industry are characterized by huge volume, high specificity of a single ship, and a high degree of customization of parts, which creates challenges for ports and shipyards and makes the assessment of how to transport blocks in a effective way more difficult [3]. Based on the characteristics of these products, the upstream suppliers of the shipbuilding industry often have wide geographical distribution and a complex logistics environment (see Table 1). The construction of high-tech ocean-going passenger ships involves a large amount of material and work, and the difficulty of the logistics of managing collection and distribution have increased exponentially. To meet these very high requirements and reduce costs, a series of management strategies must be considered.

**Table 1:** Comparison between cruise ship and commercial ship.

	Cruise ship	Commercial ship 208K BC
Construction materials	Large quantity of materials (up to 20 million parts) and facilities	The quantity of materials is small, and the number of parts is about 400,000
Distribution	distribute to all aspects of the project (such as interior project)	Focus on warehouse managementand the distribution mode is weak
Construction time	More than 10 million hours	800 thousand hours
Cable length	Approximately 2200km	Approximately 110 km
Pipeline length	Approximately 400km	22km
Suppliers	The number of suppliers exceeds 1,000 Including more than 100 strategic suppliers and nearly 900 ordinary suppliers (including more than 300 resident suppliers)	More than 10 strategic suppliers More than 400 ordinary suppliers

Among the materials and parts for assembly, intermediate products are the key basic elements in shipbuilding, and they are at the center of the problems related to poor management. The construction of the ships AIDA prima and AIDA perla by Mitsubishi Heavy Industries involved huge financial losses because of poor management in the distribution of intermediate products [4]. In particular, the personalized manufacturing of a single ship relies on intermediate product technical expertise for hull splicing. In the shipping industry, the term intermediate product refers to blocks that will be assembled at the dock after the completion of several processes, including pre-treatment, outfitting, and coating. In the construction process, there is no assembly-line production. The distribution of each component must be accurately controlled, otherwise, the entire project will be delayed. Among the many objects that need to be distributed, each ship block is of crucial importance. However, the characteristics of ship blocks, such as their heavy weight, large volume, and difficulty in handling, mean that no solution has been found to the problem of how to distribute ship blocks. Based on the principle of optimization, this paper offers an idea for suitable shipyard block transportation scheduling [5, 6].

## 2. Literature review

### 2.1 Status of research on block scheduling in workshops

Early research on the scheduling optimization of blocks was based on classical methods, such as branch and bound and integer programming. It did not include fields such as engineering science. In the 1980s and 1990s, solutions to the scheduling problem in workshops began to draw on other disciplines, and some new research means and methods emerged. Various new algorithms, such as the genetic algorithm, artificial intelligence, and the local search algorithm, were used to solve practical problems. After the 1990s, researchers began to investigate ant colony optimization and other swarm intelligence algorithms, and to apply these algorithms to their research [7].

In the shipbuilding industry, scholars began to pay more attention to this research direction during the 21st century. Cho et al. [8] studied the spatial scheduling problem of ship painting. The scheduling and spatial layout of blocks and the workload balance among working groups in the painting shop needed to be considered. Therefore, a spatial scheduling system, including operation strategy, block scheduling, arrangement, and allocation, was proposed within the constraints of the task time window, the space constraints, and the human resource constraints. The combined elements were tested based on actual scheduling data in a shipyard and were successfully applied in a painting workshop.

### 2.2 Current status of research on shipbuilding yard scheduling management

Lee et al. [9] devised a method of spatial scheduling. The construction of sections needs to occupy the site resources of the storage yard. Site resources can only be used by one section during any period of time, and they are only valuable when they are being used for blocks. The authors proposed that sections should be stacked reasonably according to their size, shape, and construction period, thereby maximizing the utilization rate of each yard and improving the value of segmented yards. Valls et al. [10] divided the scheduling problem in the yard into two stages. In the first stage, according to the basic parameters of the ship block, processing time, and process difficulty, the ship block was classified by a clustering algorithm. In the second stage, the classified group was taken as the scheduling unit, and a nonlinear model was established to find the optimal solution. However, although the mathematical method was simple, it was difficult to use to solve large-scale problems. Yan et al. [11]took irregular hull sections as their research object. A mixed IP model was established to reduce the delay time and to maximize the utilization rate of the yard, and a segmented layout rule based on standard angle filling was proposed. A solution was identified by using GA(genetic algorithm) based on segmented priority. The proposed model and algorithm were verified using data from a shipyard in China. Tao et al. [12] proposed that the execution sequence of segmented tasks has an important influence on scheduling results. Taking the number of block segments as the optimization goal, the stack location selection strategy and blocking segmented movement strategy of approach segments were implemented in a four-sided passing yard with segmented linear movement. The execution sequence of the segmented tasks was optimized by applying a tabu search. Hu et al. [13] proposed a hybrid heuristic algorithm to solve the ship block scheduling problem and they used the simulated annealing arithmetic (SA) to solve the task.

Guo et al. [14] took the average waiting time of vehicles as the optimization goal for the task execution sequence of a gantry crane. They improved the solution speed by combining a search and a backtracking algorithm. Other research, in which novel simulation models are developed to comprehensively evaluate layout design in automated terminals, finds that U-type automated terminals have the lowest energy consumption and operation costs [15]. Table 2 lists the differences between block scheduling and container scheduling.

**Table 2:** Difference between block scheduling and container scheduling.

difference	block scheduling	Container scheduling
object	Ship blocks	container
Stacking mode	Overlapping placement is not allowed	Overlapping placement is allowed
Vehicle used	flat transporter	crane
Task characteristics	Entry and exit same	Entry and exit not same

### 2.3 Status of research on scheduling between flat transporter stockyards

Ship stockyard scheduling is a vehicle scheduling problem based on the One-Vehicle and One-Cargo or Multi-Vehicle and One-Cargo transportation modes. The key to solving this problem is the assignment of transportation tasks to flat transporters and the determination of the sequence of transportation tasks executed by each flat transporter. Each task has a time window constraint. Shipbuilding enterprises generally have various types of flat transporters with different capacity restrictions, and there are other factors that complicate the scheduling problem. This problem was first raised by Joo in 2006. The minimizing of the penalty time for planned delays was the optimization goal. Joo [16] proposed a scheduling model for transporting blocks from one workshop to another under the constraints of the number of transport vehicles and the limitations of delivery times. The optimal solutions were obtained using GA and the self-adaptive algorithm, respectively. The algorithms effectiveness was evaluated using randomly generated experimental examples. Through the continuous development of these ideas, scholars have gradually formed a mature system of research on these issues.

Woon-Seek et al. [17] studied the inter-yard scheduling problem of ship blocks in a dynamic environment. A heuristic algorithm was proposed with the task time window as the main constraint. This minimized the weighted sum of no-load time and delay time. Kim et al. [18] considered that in a static environment, to solve the scheduling problem of moving different types of flat transporters between yards in shipyards, the segmented allocation strategy and the sequence strategy of transport vehicles should be determined simultaneously. A mathematical model of the optimal solution was derived to minimize the no-load transportation time and delay time. Ant colony optimization was used to arrive at an optimal scheduling scheme, and CPLEX was used to verify the feasibility of the algorithm. Shin et al. [19] suggested a transportation scheduling model considering a combination of flat transporters, thereby minimizing transportation costs and balancing the workload of each flat transporter. Three heuristic algorithms based on the tabu search method were also proposed to solve the problem, and experimental examples were provided. Chong et al. [20] identified three types of possible penalty situations. The goal was to minimize the amount of no-load transportation time, delay time, and late time.A scheduling model was established between ship subsection yards. A reasonable simplification of the model transformed the problem into the classic traveling salesman problem and the assignment problem. Then, a heuristic algorithm based on a greedy algorithm was proposed, assigning segments to available transporters and simultaneously sorting blocks for each transporter. Next, the ability of the algorithm to improve production efficiency and reduce production costs was verified. Yang et al. [21] formulated the scheduling problem of panel block construction as a multi-objective fuzzy flow shop scheduling problem with a fuzzy processing time, a fuzzy due date, and the just-in-time concept. And they used the improved genetic algorithm to solved the problem.

Roh et al. [22] examined flat transporter movement between yards, taking the minimum empty route and the interference of multiple flat transporter routes as the optimization objectives. The authors obtained the initial solution by ACO and optimized it by using GA to construct a flat transporter scheduling system, thereby solving the allocation of flat transporters in workshops and yards in shipyards. Shang et al. [23] proposed a optimization model considering the three-dimensional space, and solved the problem using GA algorithm. Finally they get a better block scheduling and improved the efficiency of the shipyard. Jawarneh [24] used adaptive bee colony optimization algorithm to solve the VRP problem with time windows. Zhang et al. [25] used adaptive large neighborhood search to solve the NP-hard problem and got a better result compared with genetic algorithm.

## 3. Methodology

### 3.1 Work process of constructing blocks

**Fig 2:**
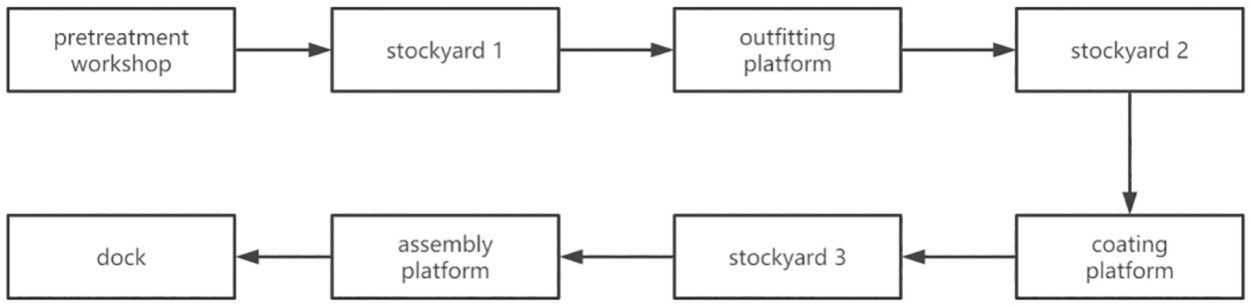
Flow process of blocks.

In the construction process, work plan requirements can mean that sections cannot always be in the same yard or workshop; they need to be transported to other yards for operation processing or temporary storage. Each yard is not only a place for the temporary storage of sections but also a place for outfitting, painting, and other processing needed for the sections. Therefore, blocks need to flow between workshops and yards (Fig 2). Thus, managing the sequence of work tasks efficiently will save money and time.

### 3.2 Model

This study takes ship blocks as the research object and aims to minimize the no-load time of flat transporter transportation under the required time constraints. It considers the construction and logistical planning of high-tech ocean-going passenger ships, the transportation capacity of the transfer vehicles, and other uncertain factors as constraints. Simulation and optimization analyses are carried out to form a reasonable and efficient intermediate product transfer plan.

#### 3.2.1 Model assumptions

The assumptions of the model are proposed below:

1. The sections loaded by flat transporter during transportation cannot exceed the load-bearing limit, and a flat transporter can only transport one section at a time.
2. Intermediate products will flow between multiple segmented yards and process workshops. Therefore, it is assumed that the positions of each yard and workshop are fixed.
3. In a distribution task, the total time is composed of the above three parts, and the loading and unloading time of sections cannot be ignored.
4. There is a task start time, and all segments can only be transported after the task is assigned. If the task has a time delay, it will incur penalties.
5. The task of a flat transporter transportation section is limited to the allowed workday hours, and all tasks need to be assigned to each flat transporter on the same day.
6. The transportation speed of the flat transporter is fixed, and the traffic conditions of the roads in the shipyard and the driving time of the flat transporter in the yard are not considered.
7. During the planning period, the shipyards demand plan for intermediate products, the number of flat transporters, the transportation speed and loading limit of vehicles, and the location of each yard and workshop are all known.
8. Many uncertain factors and accidents, such as delays caused by extreme weather and by transport vehicle failures, are not considered in the model.

#### 3.2.2 Mathematical description of the problems

It is known that there are L flat transporters and B sections to be transported. The shipping tasks for blocks contain block numbers i, the weight w_*i*_, starting position sp_*i*_; termination position ap_*i*_, task start time st_*i*_; and expected arrival time at_*i*_. The number of flat transporters is k, and the maximum load capacity of each flat transporter is ml_*k*_. The flat transporter transportation needs to meet the time window [st_*i*_, at_*i*_] (more variables are shown in Table 3). Intermediate product transfer scheduling refers to matching the flat transporter and the appropriate transportation route for the blocks to be transported, and solving the execution sequence and time of each task, thereby minimizing the down time of the flat transporter while meeting pre-arranged deadlines.

**Table 3:** The meaning of the variables in the model.

variables	Meaning
i	Number of Block
k	Number of Flat transporter
B	The total quantity of the blocks
L	The total quantity of the flat transporters
st_*i*_	Task start time of transportation block i
at_*i*_	arrival time of transporting block i
sp_*i*_	Initial position of block i at the beginning of transportation
ap_*i*_	Termination position of block i at the end of transportation
lt_*i*_	The extraction time-the time required for block i to be loaded on the flat transporter
ut_*i*_	the stacking time-The time required for block i to be unloaded from the flat transporter
*w*_*i*_	The weight of block i
ml_*i*_	Maximum load of flat transporter k
d_*ijk*_	The shortest distance between the end position of the block i and the
$d_{k}^{b}$	The distance of the flat transporter k from the parking place to the starting place of the first task
$d_{k}^{e}$	The distance from the end of the last task to the parking place of the flat transporter k
lv	Load transportation speed of flat transporter
uv	No-load transportation speed of flat transporter
α	Delay time penalty coefficient in No-load condition
β	Delay time penalty coefficient in load condition
ts_*ij*_	0–1 variable. The value is 1 when block i transports before block j, and the rest are 0
x_*i*_	Actual extraction time-the time when the transportation block i is started
y_*ik*_	0–1 variable. The value is 1 when block i is transported by flat transporter k, and the rest are 0
z_*ijk*_	0–1 variable. The value is 1 when task of block i is followed by block j, and the rest is 0

#### 3.2.3 Objective function

The sum of three functions is the objective of this section. Eq (1) is the optimization objective function considering the no-load time of flat transporters, the delay time of transportation, and the penalty for the delay time:

$$\begin{array}{c} f=min(f_{1}+f_{2}+f_{3}) \end{array}$$ (1)

Eq (2) represents the down time of the flat transporter, consisting of the time when the flat transporter moves to and from the parking place at the beginning and end of the task and the time when the flat transporter moves to the starting point of the next task after the completion of a task:

$$\begin{array}{c} f_{1}=\sum_{k=1}^{L}\frac{d_{k}^{b}+d_{k}^{e}}{uv}+\sum_{i=1}^{B}\sum_{j\neq 1,j\neq i}^{B}\sum_{k=1}^{L}\frac{z_{ijk}*d_{ijk}}{uv} \end{array}$$ (2)

Eq (3) represents the delay time penalty of the flat transporter. If the flat transporter arrives on time, there is no time penalty. If the flat transporter delivery is delayed for whatever reason, a corresponding penalty will be imposed, as follows:

$$\begin{array}{c} f_{2}=\alpha \sum_{i=1}^{B}max\{x_{i}-st_{i},0\} \end{array}$$ (3)

Eq (4) represents the time penalty for flat transporter tardiness. When the flat transporter is sent to the yard, the important task of unloading the flat transporter must be considered. Therefore, it is necessary to ensure that the time the flat transporter takes to complete the whole process does not exceed the latest time in the time frame. This delay would lead to the follow-up work being delayed. The equation is written as follows:

$$\begin{array}{c} f_{3}=\beta \sum_{i=1}^{B}max\{x_{i}+\frac{d_{ijk}}{lv}+lt_{i}+ut_{i}-at_{i},0\} \end{array}$$ (4)

#### 3.2.4 Constraint conditions

There are several constraint conditions in this model based on the actual situation. Eq (5) indicates that when the flat transporter k transports the ship block i, the weight of the block i cannot exceed the maximum load capacity of the flat transporter k:

$$\begin{array}{c} y_{ik}\cdot ml_{k}\geq w_{i} \end{array}$$ (5)

Eq (6) indicates that block i can start loading and transporting only after the task is announced. The value of is the delay time:

$$\begin{array}{c} x_{i}\geq st_{i} \end{array}$$ (6)

Eq (7) denotes the extraction time of block j when the flat transporter k transports block j after transporting block i when z_*ijk*_ = 1. The equation is written as follows:

$$\begin{array}{c} x_{j}=max\{x_{i}+\frac{d_{ijk}}{lv}+lt_{i}+ut_{i}+\frac{d_{ijk}}{uv},at_{i}\} \end{array}$$ (7)

Eq (8) indicates that the transportation task of each segment can only be assigned to one flat transporter:

$$\begin{array}{c} \sum_{k=1}^{L}y_{ik}=1 \end{array}$$ (8)

Eq (9) indicates that when there is a blocking block, the blocking block must be removed before the task can be executed; that is, when ts_*ij*_ = 1, block i must be completed before block j:

$$\begin{array}{c} x_{i}+lt_{i}\leq x_{j} \end{array}$$ (9)

Therefore, this paper derives an optimization model for the transportation of blocks. The whole equation is shown below:

$$\begin{array}{c} f=min(f_{1}+f_{2}+f_{3}) \end{array}$$ (10)

$$\begin{array}{c} f_{1}=\sum_{k=1}^{L}\frac{d_{k}^{b}+d_{k}^{e}}{uv}+\sum_{i=1}^{B}\sum_{j\neq 1,j\neq i}^{B}\sum_{k=1}^{L}\frac{z_{ijk}*d_{ijk}}{uv} \end{array}$$ (11)

$$\begin{array}{c} f_{2}=\alpha \sum_{i=1}^{B}max\{x_{i}-st_{i},0\} \end{array}$$ (12)

$$\begin{array}{c} f_{3}=\beta \sum_{i=1}^{B}max\{x_{i}+\frac{d_{ijk}}{lv}+lt_{i}+ut_{i}-at_{i},0\} \end{array}$$ (13)

$$\begin{array}{c} y_{ik}*ml_{k}\geq w_{i} \end{array}$$ (14)

$$\begin{array}{c} x_{i}\geq st_{i} \end{array}$$ (15)

$$\begin{array}{c} x_{j}=max\{x_{i}+\frac{d_{ijk}}{lv}+lt_{i}+ut_{i}+\frac{d_{ijk}}{uv},at_{i}\} \end{array}$$ (16)

$$\begin{array}{c} \sum_{k=1}^{L}y_{ik}=1 \end{array}$$ (17)

$$\begin{array}{c} x_{i}+lt_{i}\leq x_{j} \end{array}$$ (18)

### 3.3 Solving the problem using a genetic algorithm

#### 3.3.1 Shortest transportation distance

In this paper, an improved genetic algorithm is used to solve the model. First, we need to solve the transportation distance for the flat transporter in a task sequence. The method is as follows:

Step 1: Build the network of the shipyard and find the yard for blocks and the workstation. Set the shortest distance
  $$\begin{array}{c} d_{min}=\infty \end{array}$$ (19)
  and the current distance as
  $$\begin{array}{c} d_{curr}=0 \end{array}$$ (20)
Step 2: Scan the daily work plan and determine the starting and ending yards of each block. Start searching the algorithm from Yard 1.
Step 3: When the previous task has finished, calculate the distance to the next place and set the distance as d_*next*_, *thencalculate*
  $$\begin{array}{c} d_{curr}=d_{curr}+d_{next} \end{array}$$ (21)
Step 4: If d_*curr*_ < d_*min*_, set
  $$\begin{array}{c} d_{min}=d_{curr} \end{array}$$ (22)
  otherwise, the value did not change.
Step 5: Select the predecessor node of the current task node and then reselect the next workshop or yard that is different from the previous one. Return to Step 3 until all adjacent nodes of the predecessor node of the current node have been traversed.
Step 6: Acquire the optimal route and shortest distance d_*min*_.

#### 3.3.2 The genetic algorithm process

The genetic algorithm process is as follows.

**Fig 3:**
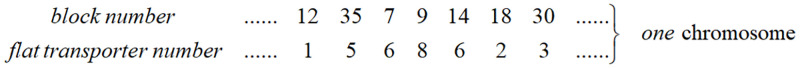
Structure of chromosome.

**Fig 4:**
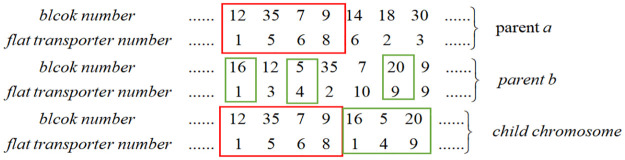
Crossover.

**Fig 5:**
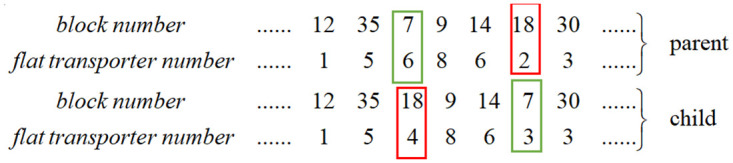
Mutation.

**Fig 6:**
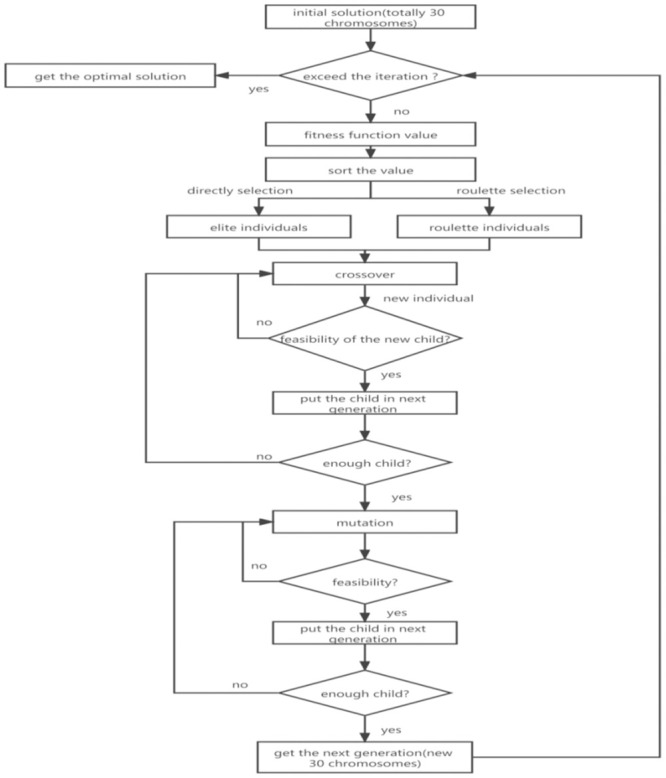
Genetic algorithm process of this paper.

Step 1: Code the task sequence chromosome. Each gene on the chromosome contains two basic information points: the task number on the daily work plan and the number of the flat transporter used to schedule the block. The chromosomes are stored in an array, from left to right, representing the order of execution. Fig 3 shows the structure of the chromosome in this study.
Step 2: Calculate the fitness function value. Step 1 obtains a number of chromosomes representing a different sequence of block scheduling. Now, we can calculate the minimum value of each chromosome using Eq (10) and the algorithm shown in Section 3.3.1. Finally, sort the value of all chromosomes using the fitness function
  $$\begin{array}{c} f^{*}=\frac{1}{f} \end{array}$$ (23)
Step 3: Through elite selection and roulette selection methods, select a certain number of individuals from the parent sample as the next-generation individuals. The proportions of the two methods are, respectively, P_*E*_ and P_*G*_
Step 4: Crossover is used to randomly set excellent individuals into pairs and to obtain a certain number of new individuals according to a probability P_*C*_ which can be considered the offspring individuals reproduced by the parents. Fig 4 illustrates the process of the crossover.
Step 5: Through mutation, certain genes of the individuals are changed according to a probability P_*V*_, and then new individuals are generated. Fig 5 shows the process of the mutation.
Step 6: Test the feasibility of the new individual. Through Steps 1–5, some new individuals will be produced, but some of them will not satisfy the constraints based on Eqs (14)–(18). Thus, we must omit the wrong one and select the correct one until the quantity of the next generation is satisfied. Fig 6 illustrates the process of the genetic algorithm.

## 4. Empirical study and results

### 4.1 Data acquisition

This case study involves actual work tasks in Shanghai Waigaoqiao shipbuilding Company in China. Although the shipyard uses the order-grabbing distribution to improve distribution efficiency, block scheduling is still chaotic and disorganized. We used the above model to optimize the distribution route to improve distribution management. The data used is from the actual block scheduling work plan of the shipyard and can be found in the data file.

#### 4.1.1 Parameters of the model based on the shipyard

**Fig 7:**
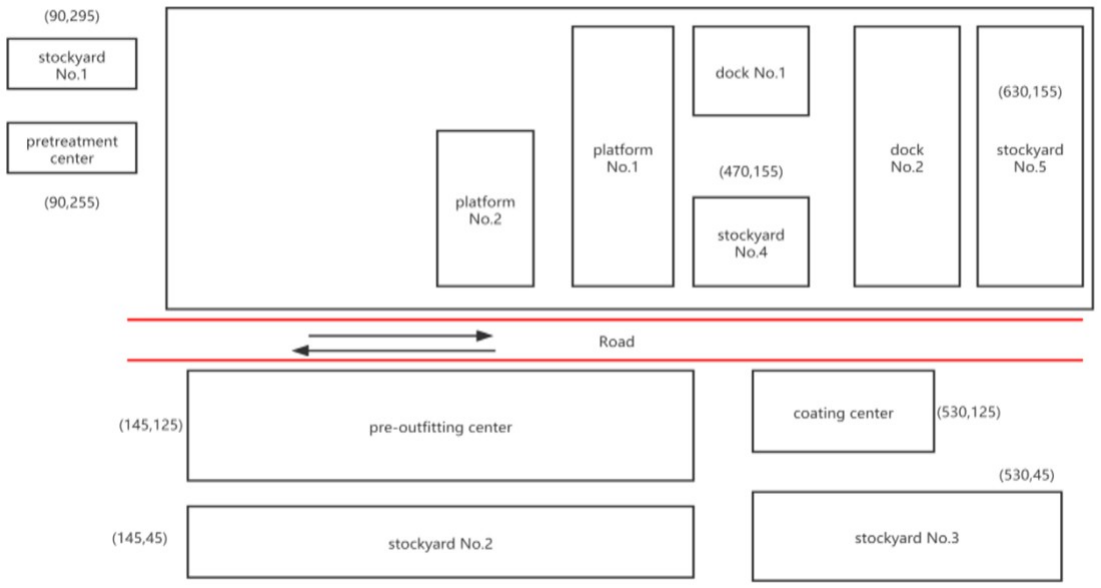
Location of the stockyard and workshops.

The following parameters are applied:

1. The planning period is weekly, the daily transportation cycle of a flat transporter is 600 mins., and the work start time is 8:00 a.m.
2. The weight of each segment is between 200 tons and 400 tons, and there are 50 transportation tasks in total.
3. There are five flat transporters (see Table 4).
4. The loading and unloading time of each section is 10 mins.
5. The running speed of a flat transporter under load is 30 meter/min., and the running speed under no load is 50 meter/min.
6. The delay penalty coefficient of the task is 5, and the delay penalty coefficient is 3.
7. The location and coordinates of the stockyard and the workshops are shown in Fig 7, and the distance between them is shown in Table 5.

**Table 4:** Info on flat transporters.

No.	Maximum capacity/T
1	250
2	270
3	320
4	380
5	420

**Table 5:** Distance among stockyards and workshops/meter.

	stockyard No.1	stockyard No.2	stockyard No.3	stockyard No.4	stockyard No.5	Pre-treatment center	Pre-outfitting center	Coating center
stockyard No.1	-	295	680	550	710	40	225	610
stockyard No.2	295	-	525	425	585	255	70	455
stockyard No.3	680	525	-	160	200	640	455	70
stockyard No.4	550	425	160	-	160	480	355	90
stockyard No.5	710	585	200	160	-	640	515	130
Pre-treatment center	40	255	640	480	640	-	185	570
Pre-outfitting center	225	70	455	355	515	185	-	385
Coating center	610	455	70	90	90	570	385	-

#### 4.1.2 Block transfer scheduling task information

This section considers the work schedule for a typical week. In practice, hundreds of work items are completed in any given week. To simplify the calculation process, we use 50 work plans to produce our model.

### 4.2 Optimization results of this case study

#### 4.2.1 The optimized route of the flat transporter based on the work schedule

Using MATLAB genetic toolbox to solve the problem, the following optimization route and task execution results were obtained (see Tables 6–10).

**Table 6:** Task execution table in day 1.

Flat transporter number	Task execution sequence	Path of the flat transporter	Total transportation time (min)	Total time (min)
4	1–3	Stockyard No.1-Pre-treatment center -stockyard No.2	15.76	55.76
5	2–4–8–9	Stockyard No.1-Pre-treatment center -stockyard No.3-stockyard No.4-coating center -stockyard No.4-coating center -Stockyard No.1	45.94	125.94
3	5–6–7–10	stockyard No.2-Pre-outfitting center -stockyard No.2-Pre-outfitting center -stockyard No.4-coating center-stockyard No.5-Stockyard No.1	40.71	120.71

**Table 7:** Task execution table in day 2.

Flat transporter number	Task execution sequence	Path of the flat transporter	Total transportation time (min)	Total time (min)
3	11–12	stockyard No.3-Pre-outfitting center -stockyard No.3-Pre-outfitting center -Stockyard No.1	57.64	97.64
5	13–16–20–21	stockyard No.3-Pre-outfitting center -Stockyard No.1-Pre-treatment center -coating center-stockyard No.5 -coating center-stockyard No.5 -Stockyard No.1	72.26	152.26
1	14–15–19	Pre-outfitting center-stockyard No.4 -Pre-outfitting center-stockyard No.4 -coating center-stockyard No.5 -Stockyard No.1	55.71	115.71
2	17–18	Stockyard No.1-Pre-treatment center -Stockyard No.1-Pre-treatment center -Stockyard No.1	4.27	24.27

**Table 8:** Task execution table in day 3.

Flat transporter number	Task execution sequence	Path of the flat transporter	Total transportation time (min)	Total time (min)
4	22–23–29	stockyard No.2-Pre-outfitting center -stockyard No.2-Pre-outfitting center -stockyard No.5-coating center-stockyard No.1	34.37	94.37
3	24–25–28	Pre-outfitting center-stockyard No.4 -Pre-outfitting center-stockyard No.4 -Pre-treatment center-stockyard No.3-stockyard No.1	79.94	139.94
2	26–27	Pre-outfitting center-stockyard No.4 -Pre-treatment center-stockyard No.3-stockyard No.1	60.98	100.98
5	30–31	stockyard No.4-coating center -stockyard No.4-coating center-stockyard No.1	31.10	71.10

**Table 9:** Task execution table in day 4.

Flat transporter number	Task execution sequence	Path of the flat transporter	Total transportation time (min)	Total time (min)
3	33–34	Pre-treatment center-stockyard No.2 -Pre-treatment center-stockyard No.2 -stockyard No.1	28.85	68.85
3	37–38	stockyard No.2-Pre-outfitting center -stockyard No.2-Pre-outfitting center -stockyard No.1	16.51	56.51
4	35–36	Coating center-stockyard No.5 -coating center-stockyard No.5 -stockyard No.1	37.78	77.78
5	32–39–40	Pre-treatment center-stockyard No.2 -Pre-outfitting center-stockyard No.2 -Pre-outfitting center-stockyard No.1	19.89	79.89

**Table 10:** Task execution table in day 5.

Flat transporter number	Task execution sequence	Path of the flat transporter	Total transportation time (min)	Total time (min)
1	44–42–45 -46–47	stockyard No.3-Pre-outfitting center -stockyard No.4-coating center -stockyard No.4-coating center -stockyard No.5-stockyard No.4 -stockyard No.1	67.45	167.45
3	41–50	Pre-outfitting center-stockyard No.4 -stockyard No.1-Pre-treatment center -stockyard No.1	33.25	73.25
5	43–48–49	Pre-outfitting center-stockyard No.4 -stockyard No.3-stockyard No.2 -stockyard No.3-stockyard No.2 -stockyard No.1	140.00	200.00

**Fig 8:**
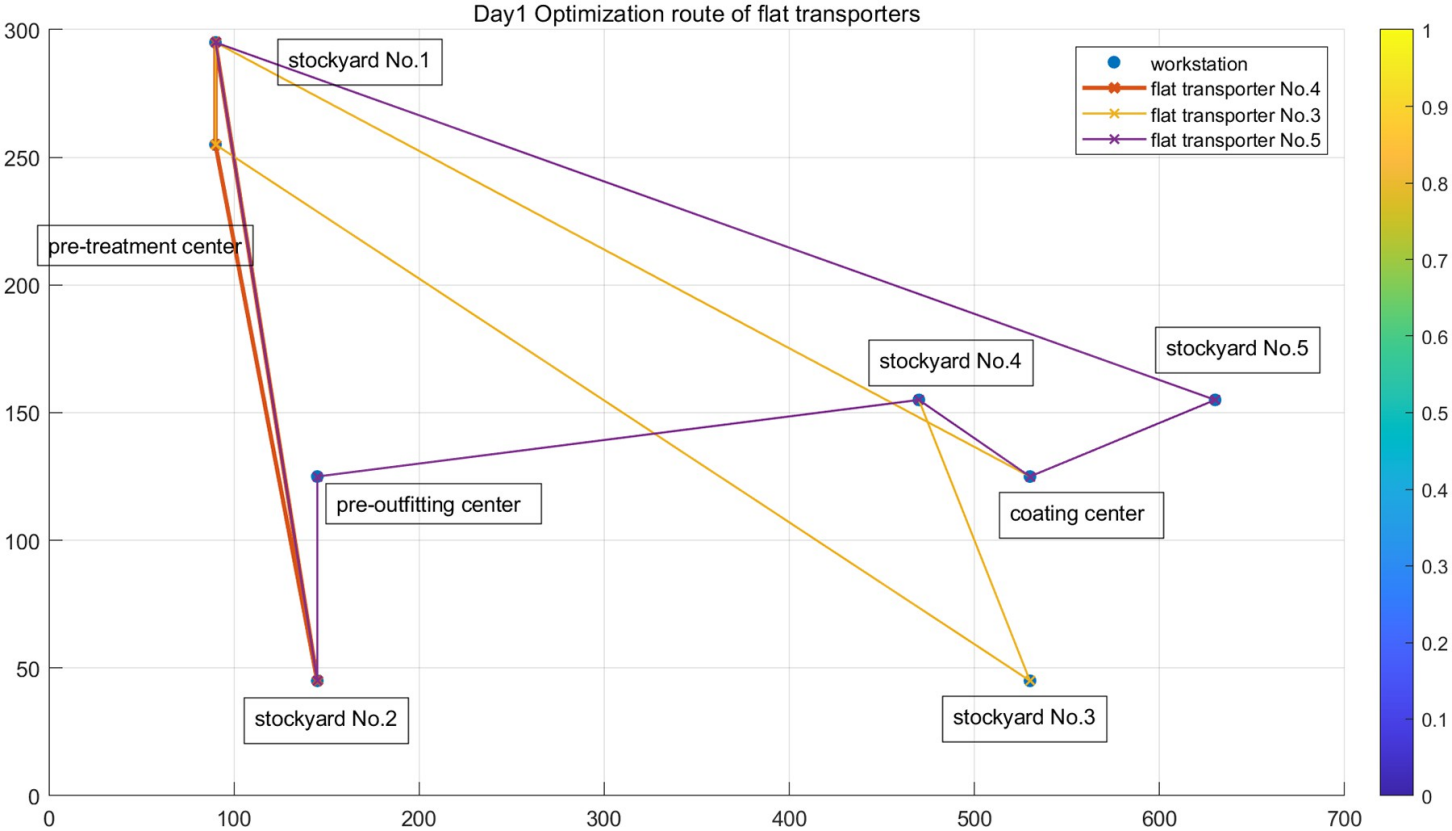
Path of a flat transporter in day 1.

The first day was taken as an example. Three flat transporters were assigned to the transportation task, and the scheduling path of each flat transporter on the first day of the planning period is shown in Fig 8. The flat transporter No. 3 started by going to the No. 2 stockyard to transport the No. 5 block to the pre-outfitting center. It then returned to the No. 2 stockyard to transport the No. 6 block to the pre-outfitting center. Finally, it went to the No. 4 stockyard to send the No. 7 block to the coating center for processing. It carried the No. 10 block in the coating center to the No. 5 stockyard, bringing the tasks of the day to an end. The loading time and road travel time amounted to 120.71 mins.

The No. 4 flat transporter completed two tasks. First, after transporting the No. 1 block to the pre-treatment center in the No. 1 stockyard, it loaded the No. 3 block and transported it to the No. 2 stockyard. The total task execution time was 55.76 mins. The No. 5 flat transporter carried out four transportation tasks. First, it transported the No. 2 block to the pre-treatment center at the No. 1 stockyard. It then transported the No. 4 block to the No. 3 stockyard pre-treatment center before driving to the No. 4 stockyard to transport the No. 8 block to the coating center. Finally, it returned to the No. 4 stockyard to transport the No. 9 block to the coating center. The total task execution time was 125.94 mins.

#### 4.2.2 Analysis of the transportation time

According to the results described in Section 4.2.1, this paper analyzes the transportation time of the optimized route of the flat transporter. First, the transportation time ratio of the flat transporter is defined as follows:

$$\begin{array}{c} t_{prop}=\frac{t_{travel}}{t_{total}}=\frac{t_{travel}}{t_{travel}+t_{load}} \end{array}$$ (24)

where t_*travel*_ refers to the travel time of the flat transporter, t_*load*_ represents the loading time of the flat transporter, and t_*total*_ represents the total execution time of the tasks.

Table 11 displays the results for the above equations. This study minimizes the no-load travel time, and distance is the main factor determining travel time. Therefore, to achieve the set goal, the transportation distance of a flat transporter will need to be as short as possible. According to the results, more than 77.78% of the tasks have a transportation time ratio of less than 50%, and the travel time accounts for a small proportion of the total time. This shows that loading time is one of the crucial reasons for the poor and ineffective management of block transportation tasks.

**Table 11:** Transportation time ratio of flat transporters.

flat transporter number	Day 1	Day 2	Day 3	Day 4	Day 5
1	-	48.15%	-	-	40.28%
2	-	17.60%	60.39%	41.90%	-
3	33.73%	59.03%	57.12%	29.22%	45.39%
4	28.26%	-	36.42%	24.90%	-
5	36.48%	47.46%	43.74%	48.57%	70.00%

Table 11 also shows that block loading is the most time-consuming part of the task. This paper obtained the optimization result without considering external interference. In practice, in shipyards, it usually takes from 30 minutes to 60 minutes for workers to load blocks. The relevant equipment and skills must be used in the loading process, and the equipment must be professionally operated. As a result, the transportation time efficiency will be lower than what Table 11 indicates. Therefore, it is essential to manage the professionals effectively. Shipyards also need to apply artificial intelligence and automation to improve transportation time efficiency.

#### 4.2.3 Utilization rate of flat transporters

Excessive use of a flat transporter will lead to the faster loss and scrapping of the flat transporter, thereby affecting work progress. This paper defines the utilization rate of a flat transporter as follows:

$$\begin{array}{c} u_{prop}=\frac{t_{total}}{t_{cycle}} \end{array}$$ (25)

where t_*total*_ refers to the total working time of the flat transporter and t_*cycle*_ represents the working cycle of 600 mins.

The results shown in Table 6 reveal that not every flat transporter engages in the task of block transportation every day. The working hours are usually concentrated in the morning or afternoon of a particular day. The highest utilization rate reaches 33.33%, the lowest is 4.05%, and the average utilization rate of all flat transporters in a week is 12.15%.

The flat transporter is the only tool that can transport blocks, and its wear rate is very high. Therefore, it often needs maintenance, making it necessary to ensure that every flat transporter is used efficiently and effectively. Table 12 shows that the utilization of flat transporters was not evenly distributed. The utilization rates of the No. 5 and No. 3 flat transporters were the highest, and the other three transporters were used less frequently. A similar process has been identified in practical work situations. It was also found that three of the flat transporters often needed repairs, while others did not require much maintenance.

**Table 12:** Utilization rate of flat transporters.

flat transporter number	Day 1	Day 2	Day 3	Day 4	Day 5
1	-	19.29%	-	-	27.91%
2	-	4.05%	16.83%	11.47%	-
3	20.12%	16.27%	23.32%	9.42%	12.21%
4	9.29%	-	15.73%	13.32%	-
5	20.99%	25.38%	11.85%	12.96%	33.33%

This paper also finds that, rather than taking the optimal solution as the final result, using the intermediate results will produce a different outcome. Although overall costs will increase, the utilization rate of the five vehicles will change clearly. Therefore, the increased cost is negatively correlated with the utilization rate of the flat transporter, resulting in an antinomic situation whereby the optimization of one objective function will change the other objective function, thereby deviating from the optimal value. Furthermore, according to the block task plan and the utilization rate of flat transporters, high-tonnage flat transporters are more durable, making it more efficient for shipbuilding enterprises to purchase more high-tonnage flat transporters (especially those over 320 tons).

#### 4.2.4 Relationship between the capacity of flat transporters and the weight of a block

This section considers the relationship between the block weight and the selection of flat transporters with different capacities, also known as the load rate. The equation is as follows:

$$\begin{array}{c} R=\frac{W_{b}}{W_{f}} \end{array}$$ (26)

where W_*b*_ represents the weight of the block, and W_*f*_ represents the weight of the selected flat transporter.

Table 13 provides the ratio of the block weight of each task to the maximum load capacity of the selected flat transporter, with an average ratio of about 87.33%. Around 50% of the tasks reached more than 90%. Therefore, the capacity of the flat transporter has been fully utilized, and, on balance, the selection of the flat transporter was reasonable.

**Table 13:** Utilization rate of flat transporters.

Task	weight/T	flat transporter/T	ratio	Task	weight/T	flat transporter/T	ratio
1	211	380	55.53%	26	237	270	87.78%
2	286	320	68.10%	27	211	270	78.15%
3	371	380	97.63%	28	286	320	89.38%
4	385	420	91.67%	29	352	380	92.63%
5	230	320	71.88%	30	399	420	95.00%
6	230	320	71.88%	31	381	420	90.71%
7	204	320	63.75%	32	345	380	90.79%
8	392	420	93.33%	33	217	270	80.37%
9	387	420	92.14%	34	268	270	99.26%
10	290	320	90.63%	35	384	420	91.43%
11	300	320	93.75%	36	390	420	92.86%
12	218	320	68.13%	37	284	320	88.75%
13	373	420	88.81%	38	306	320	95.63%
14	230	250	92.00%	39	321	380	84.47%
15	230	250	92.00%	40	338	380	88.95%
16	345	420	82.14%	41	300	320	93.75%
17	217	270	80.37%	42	218	250	87.20%
18	268	270	99.26%	43	373	420	88.81%
19	204	250	81.60%	44	206	250	82.40%
20	392	420	93.33%	45	230	250	92.00%
21	387	420	92.14%	46	230	250	92.00%
22	359	380	94.47%	47	226	250	90.40%
23	368	380	96.84%	48	395	420	94.05%
24	274	320	85.63%	49	342	420	81.43%
25	295	320	92/19%	50	286	320	89.38%

## 5 Discussion

This paper uses data from a shipyard in Shanghai as an example and constructs a model by replicating the working state of the shipyard as closely as possible. Against the background of building large-scale cruise ships in China, this paper studies block logistics in shipyards as the breakthrough point. The calculations in Section 4 show that block transportation in shipyards is difficult to manage, chiefly because the volume and weight of blocks are too large, requiring professionals to carry out loading operations before tasks can be completed. Although this paper built its model without considering the impact of personnel transfer and other abnormalities, it is still clear that the loading process is the most inefficient part of the operation. Therefore, effectively reducing the time spent loading blocks should be a key concern of future research. It is also a critical link in solving the issue of block transportation in shipyards.

This paper focuses on the stockyard and the workshop. It does not consider how to place each block in the stockyard. In fact, in shipyard work, placement of the block when it is sent to a stockyard is the next problem that should be considered. Therefore, the rationality of the work plan for the whole process should be re-examined. The author hopes to research this area next.

Finally, the significance of this paper is its use of practical cases to identify pain points in the development of shipyards. Shipyards constitute a large system, and all departments work together to form a closed loop. Only with timely feedback can problems be solved effectively and efficiently. This paper highlights problems and provides reference suggestions for the process of block transportation.

## 6 Conclusion

In this study, the minimum execution time of a block scheduling task was the objective function. The model was established using certain assumptions, and some data were used for calculation purposes. Optimized flat transporter scheduling and analysis results were obtained. Based on these findings, this paper proposes the following suggestions for the scheduling block transport on flat transporters to achieve successful management:

1. If the task of block transportation is planned in advance, the ending position of the previous task can coincide with the starting position of the next task, effectively reducing the no-load driving distance and time of flat transporters in shipbuilding enterprises and reducing the occupation of road resources, thereby increasing the possibility of performing other transportation tasks.
2. The utilization rate of flat transporters in this experiment is not high, but if a flat transporter is allowed to perform block transportation tasks in a certain period of time, more free time can be reserved for performing other tasks in shipbuilding enterprises. Situations in which flat transporters remain in a certain yard or workshop for a long time can be reduced.
3. In the practice of transportation scheduling, the penalty coefficient of task delay can be appropriately increased to reduce unfavorable situations in which the transportation task delay affects the progress of processing.
4. This model only considers a scenario in which one flat transporter transports one block. If the weight of the block carried by the flat transporter only accounts for about 50% of the maximum load capacity of the flat transporter, then a situation in which one flat transporter transports multiple blocks should be considered.

## Supporting information

S1 Data (XLSX)
